# Influence of the Inter-Trial Interval, Movement Observation, and Hand Dominance on the Previous Trial Effect

**DOI:** 10.3389/fnhum.2021.761514

**Published:** 2021-10-28

**Authors:** Hitoshi Oda, Yasushi Sawaguchi, Taku Kawasaki, Shiho Fukuda, Koichi Hiraoka

**Affiliations:** ^1^Graduate School of Comprehensive Rehabilitation, Osaka Prefecture University, Habikino, Japan; ^2^College of Health and Human Sciences, Osaka Prefecture University, Habikino, Japan

**Keywords:** previous trial effect, action observation, short-term motor memory, hand dominance, after effect

## Abstract

Previous studies have shown that current movement is influenced by the previous movement, which is known as the previous trial effect. In this study, we investigated the influence of the inter-trial interval, movement observation, and hand dominance on the previous trial effect of the non-target discrete movement. Right-handed healthy humans abducted the index finger in response to a start cue, and this task was repeated with constant inter-trial intervals. The absolute difference in the reaction time (RT) between the previous and current trials increased as the inter-trial interval increased. The absolute difference in RT reflects the reproducibility of the time taken for the motor execution between two consecutive trials. Thus, the finding supported the view that there is a carryover of movement information from one trial to the next, and that the underlying reproducibility of the RT between the two consecutive trials decays over time. This carryover of movement information is presumably conveyed by implicit short-term memory, which also decays within a short period of time. The correlation coefficient of the RT between the previous and current trials decreased with an increase in the inter-trial interval, indicating that the common responsiveness of two consecutive trials weakens over time. The absolute difference was smaller when the response was performed while observing finger movement, indicating that a carryover of the visual information to the next trial enhances the reproducibility of the motor execution process between consecutive trials. Hand dominance did not influence the absolute difference or correlation coefficient, indicating that the central process mediating previous trial effect of hand movement is not greatly lateralized.

## Introduction

The previous movement influences the next movement, called the previous trial effect (Fecteau and Munoz, 2003). This effect has been well investigated in saccadic eye movement and has also been reported even for limb movements. For example, a short-term adaptation of the previously experienced error correction to an external force load occurred within one or two trials after the previous trial of an elbow movement task (Dancause et al., 2002; Levin et al., 2010; Subramanian et al., 2018). Another study found that the initial direction and accuracy of a hitting movement was influenced by the velocity of the previous movement (de Lussanet et al., 2001, 2002), indicating that the limb movement in the previous trial influenced the limb movement in the current trial. The likely mechanism underlying the previous trial effect observed in those studies is that humans refer to previous movement information to perform the next movement (de Lussanet et al., 2002). Moreover, stride-to-stride variation of the walking speed is over-corrected in each stride (Dingwell et al., 2010; Dingwell and Cusumano, 2015). This indicates that current motor output is corrected based on the previous motor output even during rhythmic locomotor movement.

The absolute difference (AD) in the reaction time (RT) between the previous and current trials represents the reproducibility of the motor execution. The AD must be greater (i.e., reproducibility is lower) if movement information is not carried over to the next trial, as the RT in each trial is independent of that of the other trials in this case. From this perspective, the AD represents the carryover of movement information to the next trial. Reproduction of the practiced movement or force becomes inaccurate when increasing the interval between the criteria and recall trials (Adams and Dijkstra, 1966; Posner and Konick, 1966; Davis et al., 2007). Such decay of the reproduction of the target movement or force over time is considered to reflect the loss of short-term memory. Based on this view, the carryover of movement information to the next trial, which is the likely mechanism underlying the previous trial effect, must decay over time if the previous trial effect is mediated by short-term memory (hypothesis 1). If this hypothesis is true, then, the AD must increase with the inter-trial interval.

The previous trial effect has been examined in relation to index finger abduction by calculating the correlation coefficient of the motor output between the previous and current movements (Oda et al., 2021a,b). In those studies, a small but significant positive correlation of the RT or movement amplitude between the previous and current trials was found. The positive correlation of the RT between the previous and current trials represents the common process underlying the trial-to-trial variation of the motor execution shared by the two consecutive trials. The RT is dependent on responsiveness, such as arousal, attention, or prediction, in the motor system; for example, a less excitable state of the motor system leads to a longer RT, while a more excitable state leads to a reduced RT (Chen et al., 2010). Accordingly, the positive correlation in the RT between two consecutive trials observed in those previous studies represents common responsiveness shared by two consecutive trials. The responsiveness must be changed within a short-period of time. Based on this view, we hypothesized that the common responsiveness shared by consecutive trials decays over time (hypothesis 2). If this hypothesis is true, then the correlation coefficient of the RT between the two consecutive trials should decrease with an increase in the inter-trial interval.

The performance of an individual finger movement is slightly higher when vision is available (Johansson et al., 2021). The kinematics of reaching or grasping is influenced by observing movement (Jakobson and Goodale, 1991; Churchill et al., 2000; Karl et al., 2012). Observing movement (action observation) facilitates corticospinal excitability (Fadiga et al., 1995; Clark et al., 2004). More importantly, action observation helps to form motor memory (Stefan et al., 2005, 2008). Based on those findings, carryover of the visual information to the next trial, supposedly conveyed by the short-term memory, may enhance the previous trial effect (hypothesis 3). If this view is true, then the AD must be smaller when the response is performed while observing the finger movement.

Another factor that potentially influences the previous trial effect is hand dominance. In daily activity, the dominant hand is frequently used to manipulate an object, but the non-dominant hand is used for stabilizing the object (Hammond, 2002). Moreover, the cortical activity controlling the dominant hand is different from that controlling the non-dominant hand (Hammond, 2002). Thus, the hand function and neural process underlying hand movement are dependent on hand dominance. Humans likely refer to the previous movement information to achieve better motor output for the next trial. Based on this view, we hypothesized that the carryover of movement information to the next trial for dominant hand movement is different from that for the non-dominant hand movement (hypothesis 4). In this study, we tested these four hypotheses.

## Methods

### Participants

Thirteen healthy humans aged 32.9 ± 7.8 years (10 males and three females) participated in this study. The participants had no history of neurological or orthopedic disease. All participants were right-handed according to the Edinburgh Hand dominance Inventory (Oldfield, 1971). All participants gave written informed consent for study participation prior to the experiment. The experiment was approved by the Osaka Prefecture University Committee on Research Ethics (Approved number; 2020-105).

### Apparatus

**Figure 1:**
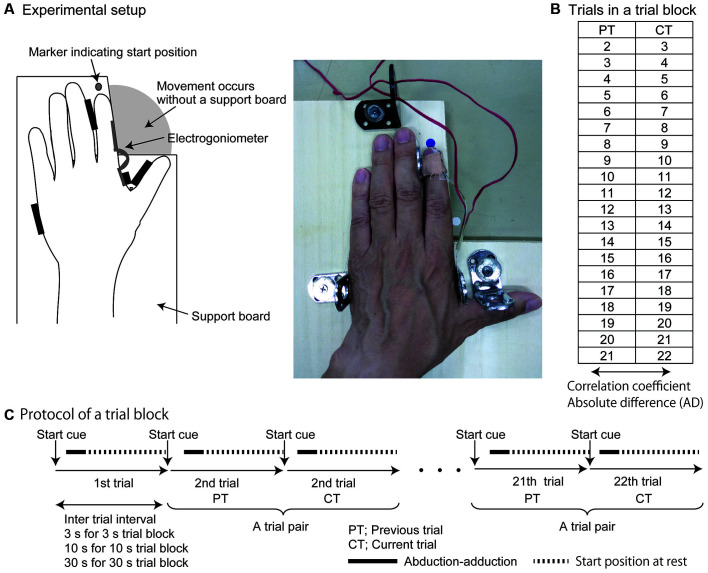
Experimental setup **(A)** analyzed trials in a trial block **(B)** and protocol of a trial block **(C)**. Numbers in **(B)** indicate the order of the trials. The first trial was not analyzed as it had no trial preceding it, as described in the “Methods” section.

The participants took a seated position in front of a table and placed their pronated forearm and hand of the tested side on a board placed over the table. The participants extended their fingers on the board at the start position (Figure 1A). The ulnar adduction of the thumb, abduction of the second and fifth metacarpal bones, and abduction of the middle finger were limited by metal plates fixed over the support board. The index finger was not restricted by the plates so that the finger was free to move. A marker was placed under the start position of the index finger. In the area in which the index finger moved, the supporting board was cut off, and thus, the finger moved freely in the air. Index finger movement (abduction and adduction of the second metacarpophalangeal joint) was measured by an electrogoniometer. Abduction of the index finger was chosen as the task because this movement is simply executed by a single joint primarily with one muscle (first dorsal interrosseous muscle) without the participation of the synergists. The electrogoniometer was made of an elastic plate on which the strain gauges (KFG-2-120-C1-11L3M2R, Kyowa Dengyo, Tokyo), indicating the joint angle, were attached. The signals from those strain gauges were amplified by a strain amplifier (DPM-751A, Kyowa Dengyo, Tokyo). The amplified analog signals from the electrogoniometer were digitized by an A/D converter (PowerLab/8sp, ADInstruments, Colorado, USA) at a 1 kHz sampling rate. Earphones that delivered an auditory start cue were placed in participants’ ears. The frequency of the auditory cue was 1 kHz.

### Procedure

A trial block testing the left or right index finger movement was conducted with or without movement observation for an inter-trial interval of 3, 10, or 30 s. Taken together, 12 trial blocks (tested side; 2 × movement observation; 2 × time interval; 3) were conducted (Table 1). The trial blocks were randomly ordered in each participant. Each trial block consisted of 22 consecutive trials (Figures 1B,C).

**Table 1:** Twelve trial blocks.

Tested hand	Interval	Visual feedback
Left hand	3 s	Eyes opened
Left hand	10 s	Eyes opened
Left hand	30 s	Eyes opened
Left hand	3 s	Eyes closed
Left hand	10 s	Eyes closed
Left hand	30 s	Eyes closed
Right hand	3 s	Eyes opened
Right hand	10 s	Eyes opened
Right hand	30 s	Eyes opened
Right hand	3 s	Eyes closed
Right hand	10 s	Eyes closed
Right hand	30 s	Eyes closed

The participants closed their eyes in the trial blocks where the movement was not observed, while they watched the index finger of the tested side in the trial blocks with movement observation. The condition of the movement observation was instructed to the participants before beginning each trial block. An experimenter monitored their eyes throughout the trial block. If they did not maintain the eyes closed or opened as instructed, the trial block was aborted and retried.

Before beginning the experiment, the participants were informed that an auditory start cue was given repeatedly with constant intervals in a trial block (Figure 1C). The beginning and end of a trial block were informed to the participants by an experimenter. Before beginning each trial, the index finger was in the relaxed state at the start position (approximately 0 degrees of abduction). The participants performed abduction followed by the adduction of the tested index finger quickly in response to an auditory start cue. The size (amplitude) of the response was not restricted. After completion of each response, the index finger was at the start position again until the next start cue was given. The experimenter monitored the finger position, and if the finger was not at the start position before the start cue, the experimenter brought their index finger back to the start position. They repeatedly performed the response to the start cue until an experimenter indicated the end of the trial block.

### Data Analysis

The movement onset of the index finger abduction was determined *via* visual inspection (Phanthanourak et al., 2016; Hiraoka et al., 2018a,b; Delahunty et al., 2019; Fortin et al., 2021; Sung et al., 2021). This analysis was conducted by a researcher in all trials across the participants. The intra-rater reliability of the visual determination of the movement onset was sufficiently high in a previous study (ICC 3, 1 = 0.992) that this analysis method was deemed reliable here (Dieterich et al., 2017). The RT, defined as the time between the start cue and the finger movement onset, was measured.

The first trial in each block was excluded from the analysis because there was no trial preceding it, which meant the first trial had a different history of movement compared with the subsequent trials. Twenty pairs of consecutive trials were organized (i.e., the second and third trials formed the first pair, the third and fourth trials formed the second pair, etc.) and then analyzed (Figure 1B). The earlier trial of each pair was the previous trial and the latter trial of the pair was the current trial. The correlation coefficient of the RT between the previous and current trials was calculated. The correlation coefficient reflects the extent of the shared common process underlying the trial-to-trial RT variation between the previous and current trials. The AD in the RT between the previous and current trials was then calculated. The AD reflects the similarity of the RT between the two consecutive trials.

A three-way ANOVA was performed to test the main effects of movement observation, inter-trial interval, and tested side (tested side; 2 × movement observation; 2 × time interval; 3). The result of Greenhouse–Geisser’s correction was reported whenever Mauchly’s test of sphericity was significant. If there was a significant main effect of the inter-trial interval, then, multiple comparison test (Bonferroni’s test) was conducted. The alpha level was set to 0.05. Excel-Toukei 2010 ver. 1.13 (Social Survey Research Information, Tokyo) was used for statistical analysis and the results are expressed as the mean and standard error of the mean.

## Results

### Mean RT

**Figure 2:**
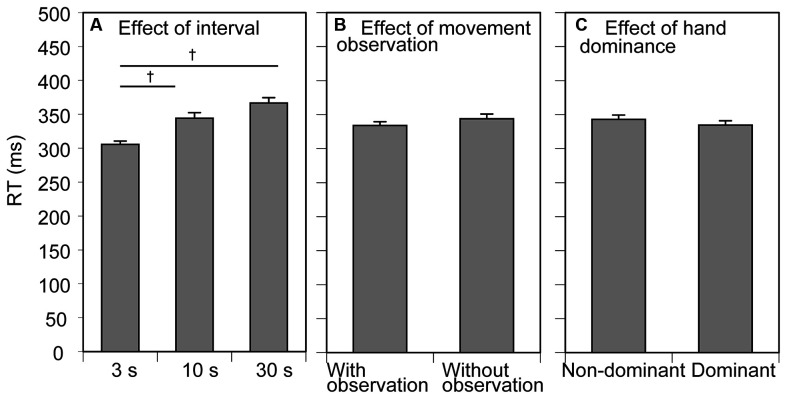
Mean reaction time (RT). Bars indicate the mean and error bars indicate the standard error of the mean. The RT averaged across the conditions of movement observation and hand dominance to show the effect of the inter-trial interval is presented in **(A)**. The RT averaged across the conditions of inter-trial interval and tested side to show the effect of movement observation is presented in **(B)**. The RT averaged across the conditions of inter-trial interval and movement observation to show the effect of hand dominance is presented in **(C)**. Daggers indicate a significant difference between the inter-trial intervals (*p* < 0.05, Bonferroni’s test).

The mean RT results for the participants are shown in Figure 2. The mean RT across the trial blocks was 339 ± 5 ms. The mean RT increased along with inter-trial interval and the inter-trial interval had a significant effect on the mean RT (*F*_(2, 24)_ = 22.658, *p* < 0.001, $\eta_{\text{p}}^{2}$ = 0.654; Figure 2A). Multiple comparison test revealed that the RT in the 3 s condition was significantly smaller than that in the 10 and 30 s conditions. There was no significant effect of movement observation (*F*_(1, 12)_ = 4.049, *p* = 0.067, *η*^2^*p* = 0.252) or tested side (*F*_(1, 12)_ = 2.930 *p* = 0.113, *η*^2^*p* = 0.196) on the mean RT (Figures 2B,C). There was no significant interaction between the movement observation and tested side (*F*_(1, 12)_ = 0.524, *p* = 0.483, *η*^2^*p* = 0.042), between the movement observation and interval (*F*_(1, 12)_ = 0.793, *p* = 0.464, *η*^2^*p* = 0.062), between the tested side and interval (*F*_(1, 12)_ = 1.412, *p* = 0.263, *η*^2^*p* = 0.105), and among the movement observation, interval, and tested side (*F*_(2, 24)_ = 0.351 *p* = 0.707, *η*^2^*p* = 0.028).

### AD

**Figure 3:**
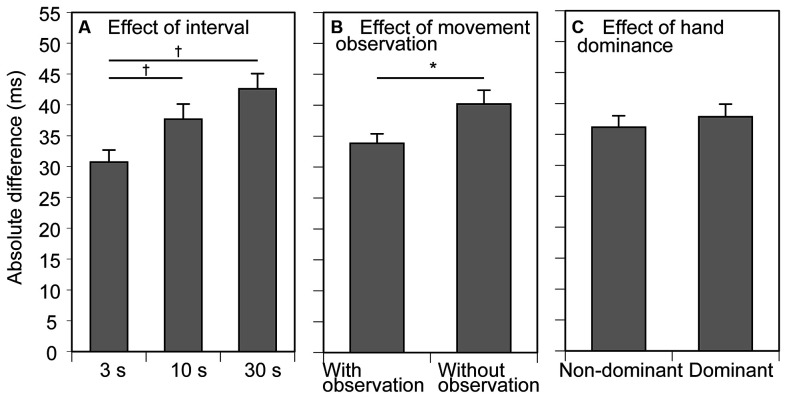
Absolute difference (AD) between the previous and current trials. Bars indicate the mean and error bars indicate the standard error of the mean. The AD averaged across the conditions of movement observation and hand dominance for showing the effect of the inter-trial interval is presented in **(A)**. The AD averaged across the conditions of the inter-trial interval and hand dominance for showing the effect of movement observation is presented in **(B)**. The AD averaged across the conditions of the inter-trial interval and movement observation for showing the effect of hand dominance is presented in **(C)**. An asterisk indicates a significant main effect of the movement observation revealed by ANOVA (*p* < 0.05). Daggers indicate a significant difference between the inter-trial intervals (*p* < 0.05, Bonferroni’s test).

The AD results for the participants are shown in Figure 3. The average AD across the trial blocks was 37 ± 1 ms. The AD increased with increase in the inter-trial interval. There was a significant main effect of inter-trial interval (*F*_(2, 24)_ = 10.020, *p* < 0.001, *η*^2^*p* = 0.455) on the AD (Figure 3A). Multiple comparison test revealed that the AD in the 3 s condition was significantly smaller than that in the 10 and 30 s conditions. The AD in the trial block with observing movement was significantly smaller than that without it (*F*_(1, 12)_ = 4.917, *p* = 0.047, *η*^2^*p* = 0.291; Figure 3B). There was no significant effect of tested side (*F*_(1, 12)_ = 0.470 *p* = 0.506, *η*^2^*p* = 0.038) on the AD (Figure 3C). There was no significant interaction between the movement observation and tested side (*F*_(1, 12)_ = 0.025, *p* = 0.878, *η*^2^*p* = 0.002), between the movement observation and interval (*F*_(1, 12)_ = 0.503, *p* = 0.611, *η*^2^*p* = 0.040), between the tested side and interval (*F*_(1, 12)_ = 1.331, *p* = 0.283, *η*^2^*p* = 0.100), and among the movement observation, interval, and tested side (*F*_(2, 24)_ = 1.386, *p* = 0.269, *η*^2^*p* = 0.104).

### Correlation Coefficient

**Figure 4:**
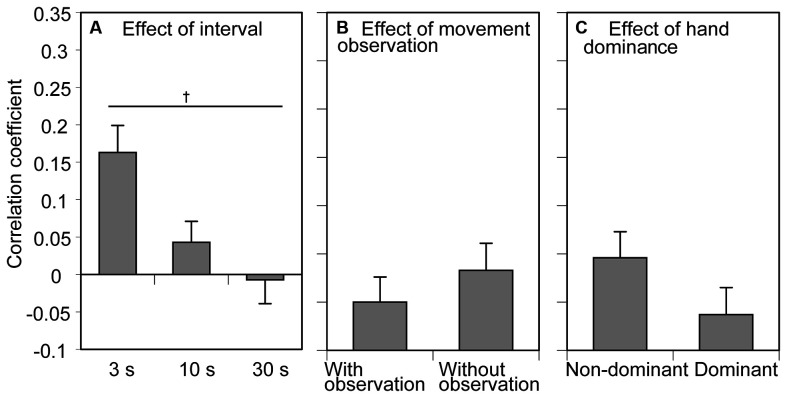
The correlation coefficient of the reaction time (RT) between the previous and current movement trials. Bars show the mean and error bars are the standard error of the mean. The correlation coefficient averaged across the conditions of movement observation and hand dominance to show the effect of the inter-trial interval is presented in **(A)**. The correlation coefficient averaged across the conditions of inter-trial interval and tested side to show the effect of movement observation is presented in **(B)**. The correlation coefficient averaged across the conditions of inter-trial interval and movement observation to show the effect of hand dominance is presented in **(C)**. A dagger indicates a significant difference between the inter-trial intervals (*p* < 0.05, Bonferroni’s test).

The mean correlation coefficient of the RT between the previous and current trials across the participants is shown in Figure 4. There was a significant effect of the inter-trial interval on the correlation coefficient (*F*_(2, 24)_ = 5.663, *p* = 0.010, *η*^2^*p* = 0.321; Figure 4A). The correlation coefficient was positive and tended to be small as the inter-trial interval increased. Multiple comparison test revealed that the correlation coefficient in the 3 s condition was significantly greater than that in the 30 s conditon. There was no significant main effect of movement observation (*F*_(1, 12)_ = 0.975, *p* = 0.343, *η*^2^*p* = 0.075) or tested side (*F*_(1, 12)_ = 3.983, *p* = 0.069, *η*^2^*p* = 0.249) on the correlation coefficient (Figures 4B,C). The average correlation coefficient between the previous and current tasks in the 30 s task indicated negative value, but the value was very small (i.e., −0.007). Thus, this negativity was negligible. There was no significant interaction between the movement observation and tested side (*F*_(1, 12)_ = 4.072, *p* = 0.067, *η*^2^*p* = 0.253), between the movement observation and interval (*F*_(1, 12)_ = 1.073, *p* = 0.358, *η*^2^*p* = 0.082), between the tested side and interval (*F*_(1, 12)_ = 0.238, *p* = 0.790, *η*^2^*p* = 0.019), and among the movement observation, interval, and tested side (*F*_(2, 24)_ = 0.830, *p* = 0.448, *η*^2^*p* = 0.065).

## Discussion

### Time Prediction and Inter-Trial Interval

The correlation coefficient of the RT between the previous and current trials decreased as the inter-trial interval increased. The positive correlation of the RT between the previous and current trials reflects the common mechanism underlying the trial-to-trial RT variation shared by the previous and current trials. This variation is not derived from the task-specific effect, as the task was the same across trials (i.e., index finger abduction followed by adduction in response to a start cue). Rather, this must have come from between-trial variability in responsiveness, such as attention, arousal, or prediction, influencing the RT (Eason et al., 1969). Thus, our finding can be explained by hypothesis two that the common responsiveness shared by two consecutive trials decayed over time.

Time prediction may be the mechanism underlying common responsiveness shared by the previous and current trials. The simple RT was prolonged by increasing the foreperiod (time between the warning and start cues; Klemmer, 1957; Niemi and Näätänen, 1981). This is explained by the view that predicting the time of the start cue becomes more difficult when this period is longer. In the present study, the start cue of the previous trial must have played a role in the warning cue that enabled the participants to predict the time of the start cue in the next trial, because the inter-trial interval was constant in a trial block. Therefore, a longer inter-trial interval in the present study is the situation similar to a longer foreperiod of the warned RT task. Accordingly, time prediction is more difficult when the inter-trial interval is longer. Thus, the decrease in the correlation coefficient with an increase in the inter-trial interval may be explained by difficult time prediction of the next start cue causing weak common time prediction process between the two consecutive trials for the longer inter-trial interval.

### Short-Term Memory and Inter-Trial Interval

The AD must be greater if the previous movement information is not carried over to the next trial, because the RT for each trial would be independent of the other trials if referring to the previous movement information is not available. Based on this view, the AD likely represents the carryover of movement information to the next trial. The AD increased as the inter-trial interval increased. This means that the carryover of movement information from one trial to the next weakens as the inter-trial interval between two consecutive trials increases.

Rats are able to remember the response of the previous trial (Church, 1980), indicating that they retrieve and use short-term memory of the previous trial in the inter-trial interval. The reproduction of the practiced movement becomes less accurate as the interval between the criteria and recall trials increases (Adams and Dijkstra, 1966; Posner and Konick, 1966; Davis et al., 2007). Those previous findings indicate that short-term memory is lost over time.

Losing short-term memory over time likely occurs in the inter-trial interval of the force production task. The voluntary force decreases over time after removal of the visual target force feedback when one attempts to maintain the target force level (unintentional force drift; Vaillancourt and Russell, 2002; Ambike et al., 2015; Neely et al., 2017; Solnik et al., 2017). Unintentional force drift is thought to be due to the loss of short-term memory; i.e., memory hypothesis (Vaillancourt and Russell, 2002; Cowan, 2008; Neely et al., 2017). In a previous study, unintentional force drift began 0.5–1.5 s after the removal of visual feedback (Vaillancourt and Russell, 2002). With the force reproduction task, the practiced movement or force was forgotten within 30 s (Posner and Konick, 1966; Davis et al., 2007). Those time scales of the force decay or forgetting the movement or force fit well with the time scale of the decay in AD observed in the present study. Taken together, the decay of the previous trial effect over time, represented by the increase in the AD along with inter-trial interval, can be explained by the loss of short-term memory over time. Therefore, hypothesis one was supported.

It is thought that the previous trial effect is mediated by implicit short-term memory (Henson, 2003). In the present study, non-target simple movement was repeated in response to a start cue. Thus, in the task conducted in the present study, the participants did not memorize the process of the previous motor task explicitly. Accordingly, short-term memory, which mediates the previous trial effect, must be implicitly processed. Taken together, carryover of the previous movement information to the next during the non-target discrete movement is likely conveyed by implicit short-term memory.

A recent study revalidated the memory hypothesis that unintentional force drift is due to the loss of short-term memory over time (Solnik et al., 2017). This was achieved by making a comparison between the tonic continuous force output and tonic force output with the inter-phase interval (interval between tonic force output phases). The motor command and somatosensation are absent in the inter-phase interval, causing difficulty in holding short-term memory. Based on this view, they hypothesized that unintentional force drift is greater when the inter-phase interval is present if this drift is explained by the loss of short-term memory. The force decreased over time after removal of the target force feedback during the continuous force output, but such a decrease did not occur when the intervals were inserted between the tonic force outputs. Based on this finding, the authors of this study did not support the memory hypothesis but proposed their original hypothesis; the control of motor actions with changes in referent coordinates for participating effectors.

Accordingly, one may speculate that this previous conflicts with our interpretation that the increase in the AD with increase in the inter-trial interval is attributed to the loss of short-term memory over time. However, the finding and hypothesis by Solnik and colleagues may not be applicable to our present finding, because the task in the present study was ballistic abduction of the index finger without a target, but the task conducted in the previous study was the reproduction of the tonic target force output. The mechanism underlying the target force output task and that underlying the discrete (ballistic) movement without a target may not be compatible.

The decay in the verbal performance over time is interpreted not only by the loss of short-term memory but also by interference in the retention phase for recall (Jonides et al., 2008). Cowan suggested that rehearsal, retrieving long-term memory, or temporal distinctiveness in the retention phase for recall may interfere with the maintenance of the performance in the retention phase causing the decay of the performance over time (Cowan, 2008). This may be a possible alternative explanation for the increase in the AD with an increase in the inter-trial interval rather than the loss of short-term memory over time.

### Observing Movement

The AD was significantly smaller when the participants observed the tested finger movement. Observing the finger movement in the current trial did not influence the RT in the current trial, because the motor response is absent in the RT (i.e., the period between the start cue and response onset). Rather, visual information of the finger movement in the previous trial is carried over to the next trial by observing the finger movement, that is, the effect of observing the finger movement on the AD represents the carryover of visual information regarding the motor response from one trial to the next. Thus, our findings support hypothesis 3 that carryover of visual information from one trial to the next can be achieved by observing the movement and that this enhances the reproducibility of the motor execution process over two consecutive trials.

When participants performed movement, they must have stored the memory of the motor command and somatosensation. When they performed the movement while observing movement of the finger, participants must have stored memory of not only the motor command and somatosensation but also visual information of the movement. Previous studies have shown that action observation forms motor memory (Stefan et al., 2005, 2008). Thus, our finding is explained by the view that carryover of visual information from one trial to the next enhances the reproducibility of the motor execution process and is conveyed by implicit short-term memory. Further studies are needed to confirm this hypothetical view.

### Hand Dominance

We hypothesized that the carryover of movement information to the next trial was dependent on hand dominance (hypothesis 4). However, there was no significant effect of hand dominance on either the AD or the correlation coefficient between the previous and current trials. This indicates that hand dominance does not influence the previous trial effect, mediated by the shared common process underlying the trial-to-trial RT variation or carryover of movement information to the next trial. Hypothesis 4 was therefore not supported.

It has been reported that the hand function and cortical control of the dominant hand are different from that of the non-dominant hand (Hammond, 2002). Nevertheless, previous studies reported no significant difference in the RT of the first dorsal interosseous muscle response between response sides either in the simple or choice RT task (Hiraoka et al., 2018a,b). This previous finding supports the view that the time taken for the motor execution is not significantly different between the dominant and non-dominant hands. In the present study, the time taken for the motor execution was measured by the RT. Thus, our findings indicated that, as with no great difference in the time taken for the motor execution between the hands in the previous findings, carryover of the movement information to the next trial and common responsiveness shared by the consecutive two trials, particularly represented by the RT, are not greatly different between the dominant and non-dominant hands.

A previous study has shown that the posterior parietal cortex is the site contributing to the sensory-stimulus history (Akrami et al., 2018). More importantly, the supplementary motor area (SMA) influences the correlation coefficient of the RT between the previous and current movements (Oda et al., 2021a). There is an asymmetrical activity of the SMA during unimanual movement; left SMA is equally active during the movement of either hand, while right SMA is more active for left-hand movement (Rogers et al., 2004). In spite of those previous findings, there is no evidence showing the asymmetrical contribution of the SMA on the previous trial effect. Taken together, the most plausible explanation for the absent influence of hand dominance on the previous trial effect in the present study is that the central process of the previous trial effect is not greatly lateralized.

## Conclusions

The AD in the RT between the previous and current trials increased with longer inter-trial intervals, indicating that the carryover of movement information from one trial to the next decayed over time. This supports the view that implicit short-term memory conveys previous movement information to the next trial during the non-target discrete movements. The correlation coefficient of the RT between the previous and current trials decreased with an increase in the inter-trial interval, indicating that the common responsiveness shared by the two consecutive trials weakened over time. The AD was smaller when the response was performed while observing finger movement, indicating that there is a carryover of visual information from one trial to the next conveyed by implicit short-term memory, which enhances the reproducibility of the time taken for the motor execution between two consecutive trials.

## Data Availability Statement

The raw data supporting the conclusions of this article will be made available by the authors, without undue reservation.

## Ethics Statement

The studies involving human participants were reviewed and approved by Osaka Prefecture University Committee on Research Ethics. The patients/participants provided their written informed consent to participate in this study.

## Author Contributions

All authors listed have made a substantial, direct and intellectual contribution to the work, and approved it for publication. All authors contributed to the article and approved the submitted version.

## Conflict of Interest

The authors declare that the research was conducted in the absence of any commercial or financial relationships that could be construed as a potential conflict of interest.

## Publisher’s Note

All claims expressed in this article are solely those of the authors and do not necessarily represent those of their affiliated organizations, or those of the publisher, the editors and the reviewers. Any product that may be evaluated in this article, or claim that may be made by its manufacturer, is not guaranteed or endorsed by the publisher.
